# Construction of *in vitro* 3-D model for lung cancer-cell metastasis study

**DOI:** 10.1186/s12885-022-09546-9

**Published:** 2022-04-21

**Authors:** Rongrong Jiang, Jiechun Huang, Xiaotian Sun, Xianglin Chu, Fangrui Wang, Jie Zhou, Qihui Fan, Liewen Pang

**Affiliations:** 1 Department of Cardiothoracic Surgery, Huashan Hospital, Fudan University, Shanghai, P.R. China; 2 Qibao Community Health Service Center, Shanghai, P.R. China; 3 Beijing National Laboratory for Condensed Matter Physics and Laboratory of Soft Matter and Biological Physics, Institute of Physics, Chinese Academy of Sciences, Beijing, China

**Keywords:** 3-D model, Collagen hydrogel, Cancer metastasis, Drug screening, Microfluidic system

## Abstract

**Background:**

Cancer metastasis is the main cause of mortality in cancer patients. However, the drugs targeting metastasis processes are still lacking, which is partially due to the short of effective *in vitro* model for cell invasion studies. The traditional 2-D culture method cannot reveal the interaction between cells and the surrounding extracellular matrix during invasion process, while the animal models usually are too complex to explain mechanisms in detail. Therefore, a precise and efficient 3-D *in vitro* model is highly desirable for cell invasion studies and drug screening tests.

**Methods:**

Precise micro-fabrication techniques are developed and integrated with soft hydrogels for constructing of 3-D lung-cancer micro-environment, mimicking the pulmonary gland or alveoli as *in vivo*.

**Results:**

A 3-D *in vitro* model for cancer cell culture and metastasis studies is developed with advanced micro-fabrication technique, combining microfluidic system with soft hydrogel. The constructed microfluidic platform can provide nutrition and bio-chemical factors in a continuous transportation mode and has the potential to form stable chemical gradient for cancer invasion research. Hundreds of micro-chamber arrays are constructed within the collagen gel, ensuring that all surrounding substrates for tumor cells are composed of natural collagen hydrogel, like the *in vivo* micro-environment. The 3-D *in vitro* model can also provide a fully transparent platform for the visual observation of the cell morphology, proliferation, invasion, cell-assembly, and even the protein expression by immune-fluorescent tests if needed.

The lung-cancer cells A549 and normal lung epithelial cells (HPAEpiCs) have been seeded into the 3-D system. It is found out that cells can normally proliferate in the microwells for a long period. Moreover, although the cancer cells A549 and alveolar epithelial cells HPAEpiCs have the similar morphology on 2-D solid substrate, in the 3-D system the cancer cells A549 distributed sparsely as single round cells on the extracellular matrix (ECM) when they attached to the substrate, while the normal lung epithelial cells can form cell aggregates, like the structure of normal tissue. Importantly, cancer cells cultured in the 3-D *in vitro* model can exhibit the interaction between cells and extracellular matrix. As shown in the confocal microscope images, the A549 cells present round and isolated morphology without much invasion into ECM, while starting from around Day 5, cells changed their shape to be spindle-like, as in mesenchymal morphology, and then started to destroy the surrounding ECM and invade out of the micro-chambers.

**Conclusions:**

A 3-D *in vitro* model is constructed for cancer cell invasion studies, combining the microfluidic system and micro-chamber structures within hydrogel. To show the invasion process of lung cancer cells, the cell morphology, proliferation, and invasion process are all analyzed. The results confirmed that the micro-environment in the 3-D model is vital for revealing the lung cancer cell invasion as *in vivo*.

**Supplementary Information:**

The online version contains supplementary material available at 10.1186/s12885-022-09546-9.

## Introduction

Cancer metastasis is composed of complex processes, including decreased cell–cell adhesion, increased cell motility, invasion of cancer cells into surrounding tissues, and finally the colonization and formation of secondary tumors [1–3]. The invasion of cancer cells is very hard to be controlled, thus cancer metastasis is the most lethal factor for tumor patients, especially for lung cancer patients [2, 4]. Currently, there is few drug targeting the molecules in metastasis processes, partially due to the lacking of effective *in vitro* model, which can mimic cancer cells invasion as *in vivo* [1, 5]. The traditional 2-D culture system cannot reveal the interaction between cells and the surrounding extracellular matrix (ECM) [6–8], while the animal models usually are too complex to explore mechanisms in detail. Therefore, it is important to find an effective *in vitro* model to study the invasion mechanism and also to quantify the invasion phenomena, which is important for providing a precise evaluation platform for clinical therapy or drug screening [9–12].

There have been some *in vitro* models for cancer invasion studies. The fundamental method is on 2-D substrate, called wound healing assay or scratch assay. In this method, a wound was made by scratching the cell monolayer in a straight line with a sterile pipette tip. Then cell migration toward the wound center would be monitored, and the cell migration speed is analyzed to indicate the cell-invasion ability. This method is easy for handling and can present cell migration results. However, it can only show the cell migration ability, but not exactly the ability of cell invasion through the surrounding matrix as *in vivo* [13]. Therefore, it is still a partial way to evaluate cell invasion. Another popular quasi-3D method for invasion studies is the Boyden chamber (Transwell) assay. Cells are separated in two neighboring chambers by a thin layer of porous material. Cells can squeeze through or digest the thin porous layer, and migrate to another chamber, like the cell invasion into a distant region [14]. However, the separating 2-D layer is not like the ECM of the real tissue, thus lacking of the ability for precisely revealing the metastasis process [5, 15].

A lot of microfluidic models have been constructed to study the lung cancer cells, e.g., the designed microfluidic chip with multi-chambers to assist the formation of cancer cell spheroids [16], and the chip to assist the co-culture of lung cancer cells and cancer-associated fibroblasts [17]. However, these models only provide 2-D culture substrates for cells, lacking of the ECM, thus they cannot reveal the important interactions between cancer cells and surrounding ECM during invasion.

Some 3-D *in vitro* microfluidic models also emerge in recent years, which contain the hydrogels as ECM for cell culture. A common method is to mix the isolated cancer cells or pre-formed spheroids within the hydrogels [18], which can present the interaction between cells and ECM, but cannot fully recapitulate the tissue structures, such as the gland and alveoli. To mimic the interface within the gland *in vivo*, a 3-D microfluidic model has been developed in the previous work [9, 19]. Generally, a microfluidic system fabricated with biocompatible polydimethylsiloxane (PDMS) is composed with central hydrogel platform and four micro-channels surrounding the platform [20, 21]. Natural hydrogel type I collagen was filled in the central platform of the 3-D model as ECM for culturing tumor cells, because collagen is the most abundant ECM component in human tissues [22]. Especially, hundreds of micro-chambers were fabricated within the Collagen gel, mimicking the structure of gland as *in vivo*. The hydrogel platform with micro-chambers is the pivotal component in the 3-D model because it mimics the ECM structures of the tumor microenvironment and provides high-throughput units for tests [6, 23, 24].

However, collagen hydrogel is also very soft and fragile, composed of more than 90% of water [25]. Therefore, it is very hard to construct micro-features on the soft collagen gel. In previous studies, the collagen hydrogels integrated in the microfluidic system usually are with high concentrations of more than 6 mg/ml, to ensure the hydrogel stiffness and strength, avoiding being damaged during the fabrication process. However, ECM *in vivo* usually has much lower stiffness than that of the 6 mg/ml collagen gel, especially in certain soft tissues, such as the lung gland and alveoli. If the ECM stiffness is too high, it will be impossible for observing the cancer cells invading into the hydrogel and starting the metastasis. It is likely that lung cancer cells would be confined within the micro-chambers without any invasion, like other normal cells. Or it will take too long time for invasion studies if lung cancer cells are confined in collagen micro-chambers with such high stiffness. To mimic the softer micro-environment and allow the lung cancer cell culture and invasion, we revised the micro-structure of the microfluidic chip to provide enough support for low concentration collagen hydrogel. Meanwhile, we optimized the soft micro-fabrication technology for molding collagen in such a low concentration. After the construction of the 3-D model, cells are seeded in these micro-chambers in the softer collagen for further invasion study. Within the *in vitro* 3-D model with softer ECM, which is compatible to lung tissue stiffness, lung cancer cell invasion is characterized and studied. Meanwhile, due to the transparency of the 3-D model, the cell morphology, proliferation, invasion, cell-assembly, and even the bio-markers can be analyzed within it, providing a promising platform for cell invasion studies. The results are quantified by various methods, showing that lung cancer cells and alveolar epithelial cells can normally proliferate within the micro-chambers with their unique morphology as *in vivo*. Also, it can be seen that lung cancer cells present isolated round shape during the first days in micro-chambers, and then cell morphology changed to mesenchymal type and started to destroy the ECM and invaded out of the micro-chambers rapidly. These phenomena all reveal that the tumor micro-environment in the constructed *in vitro* model is more like that as *in vivo*.

## Materials and methods

### Cell culture

A non-small-cell lung carcinoma (NSCLC) cell line A549, obtained from China Infrastructure of Cell Line Resources (Beijing, China), was adopted for characterization of the 3-D *in vitro* model. A549 was cultured in McCoy’s 5A medium (Corning) with 10% FBS (Gibco) and 1% penicillin/streptomycin (Corning). Green fluorescent protein (GFP) was expressed in cells by lentivirus transduction to construct A549-GFP cell line. Immortalized human alveolar epithelial cells (HPAEpiCs), generated from Type II pneumocytes of human lung tissue, were cultured with RPMI 1640 medium (Corning) supplemented with 10% FBS and 1% penicillin/streptomycin (Corning), as the normal lung epithelial cell model compared to A549 cancer cells. HPAEpiCs were stained with Calcein AM (Donjindo) for fluorescent imaging.

### Chip microfabrication for the *in vitro* 3-D model

The pattern of microfluidic chip of the *in vitro* 3-D model was designed with L-edit software (Tanner EDA), and printed onto a chrome mask using a laser writer with high precision (Heidelberg DWL2000 Mask Writer). Silicon wafers with 500-μm thickness were cleaned and spin-coated with 4.0-μm positive photoresist (ECL3027). The size for each micro-chamber on stamps is 200 × 200 μm with depth of 100 μm. The photoresist pattern was created by casting the mask with standard UV lithography techniques. After the exposure, the residual photoresist on the silicon wafer was removed in the RIE chamber (Reactive Ion Etching, Plasmalab System 100) with O_2_ plasma. Then the DRIE (Deep Reactive Ion Etching, Plasmalab System 100) Bosch technique was utilized to etch the exposed silicon regions, till the thickness of 250 μm. After etching, the photoresist was removed with acetone, and subsequently with a Piranha solution.

A biocompatible polymer PDMS was used for fabricating the microfluidic chip. To prepare the PDMS, a Sylgard gel and elastomer (Dow Corning) were thoroughly blended at a ratio of 10: 1 and then was degassed in a vacuum chamber. The prepared liquid mixture was poured onto the silicon mask with microfluidic feature. Then after degassing, the mixture on silicon mask was put in the 60 °C oven for 3 h to crosslink and form the solid PDMS. The PDMS stamps for fabricating the micro-wells in the collagen hydrogel are made with the same method but casted on silicon mold with different patterns. The solidified PDMS pieces with patterns were peeled off from the silicon mold and sterilized with 75% ethanol before using. The fabricated PDMS substrate was coated with 1 mg/ml poly-D-lysine (PDL) solution (Sigma) at 37 °C for 12 h to increase the adhesion with collagen hydrogel. The PDMS stamps with pre-defined micro-well features were coated with 2% Pluronic F127 (Thermo Fisher) solution for detaching from collagen gel. The fabrication processes are also generalized in Fig. 2.

### Assembly of the *in vitro* 3-D cell culture model

Type I collagen (high concentration, 354,249, Corning) stock solution was diluted and neutralized to 2 mg/mL with buffer solutions of 10 × PBS, sterile deionized water, and 1 mol/L NaOH (Fluka). These processes were all handled on ice. The PDMS stamps were rinsed three times with diluted collagen, and were inverted to cover the PDMS substrate with pre-filled collagen solution. Meanwhile, a thin layer of collagen was prepared by evenly spreading collagen solution on a cover glass, which served as the collagen cover for micro-wells. The PDMS chips and stamps with collagen solution were incubated at 37 °C for 30 min to be gelled. Then the PDMS stamp was carefully detached from the PDMS substrate and collagen gel to form the micro-wells. 30 μl cell suspension with density of 1 × 10^7^ cells/ml was added onto the collagen area, and the chip was gently shaken to ensure most of the cells dropped into the micro-wells. The cell-seeded chip was incubated at 37 °C for 1 h, allowing cells to firmly attach to the collagen. At last, the collagen layer was covered onto the collagen substrate to form the sealed micro-chambers. A Plexiglas jig was designed to clamp the chip for sealing. There are medium-well on the inlets of the medium channels for cell culture and adding of other bio-factors if needed. Cell culture medium can also be supplied by the pump system with constant pressure to control the flow rate.

### Imaging and data analysis

The assembled 3-D model was mount onto the stage of inverted fluorescent microscope, which was equipped with an on-stage incubator to ensure the constant temperature and CO_2_ for the cell culture. Cells and collagen gel in the 3-D model were also imaged with a confocal laser scanning microscope (CLSM, Leica SP8) with 25 × WATER immersion objective. The pinhole is set to 1.0 airy unit, and a PMT detector collects the light signals. Refection mode of the CLSM was used to image the collagen fibers, as in previous studies [26]. All images were captured with the same microscopic setting parameters to keep consistence for further image analysis. The images were analyzed with an open-source software ImageJ. Around 91 cells were analyzed in parallel tests. The error bars indicate the SEM in each group.

## Results and discussion

### Construction of the 3-D in vitro model with low-concentration collagen hydrogel

The collagen hydrogel is very soft and fragile, thus the difficulty for micro-fabrication is very high. To build a microfluidic platform as a controllable *in vitro* 3-D model for advanced cell culture and cancer cell invasion study, we tried to construct micro-chambers within collagen hydrogels to mimic the structure of the softer tissues, such as pulmonary gland or alveoli. Alveoli are an important part in lung, which are tiny balloon-shaped structures. Lung cancer usually starts from alveoli when a mass of cells grows out of control [27]. Therefore, it is important to mimic the alveoli micro-structures *in vitro* for lung cancer invasion studies. However, the current hydrogel micro-molding technology within micro-fluidic chips usually works on the hydrogels with high stiffness, such as the collagen I hydrogel with protein concentration of higher than 6 mg/ml. Once the hydrogel is too soft, it is too fragile to be constructed, especially on low aspect ratio shape. Hydrogels would be detached or removed, or the micro-feature would be damaged during the stamp peeling off process. But this stiffness is usually much higher than the tissue *in vivo*, especially the soft lung tissue.

It would be not proper to use the high-stiffness hydrogel to fabricate micro-chambers for lung cancer cell invasion study, because the tissues around lung cancer cells are usually very soft *in vivo*. Once the hard hydrogel was used, the lung cancer cells might be limited to these stiff materials without invasion or may take too much time and prolong the effective study period. Therefore, a lot of effort is taken in developing the micro-molding technology on soft hydrogels. Now the 2 mg/ml collagen hydrogel micro-molding can be realized by enlarging and modifying the micro-fluidic chip structures and optimizing the surface coating process with Pluoronic F-127 to assist removing the stamp without damaging the micro-features on relative large-scale collagen platform.

**Fig. 1:**
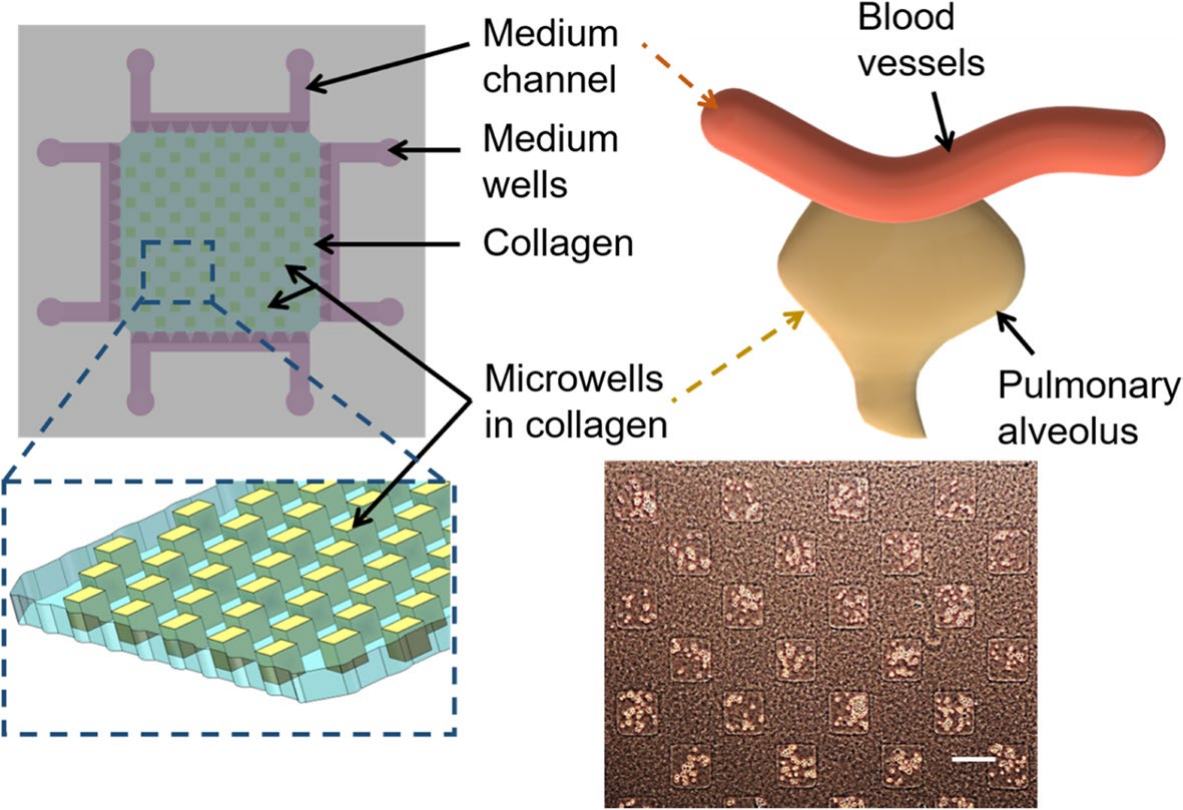
Schematic images of the 3-D microfluidic system for *in vitro* culture model. Hundreds of micro-chambers are constructed within the collagen hydrogel for mimicking the pulmonary gland or alveoli. Bottom right image shows the cells cultured within the micro-chamber arrays. Scale bar is 200 μm

**Fig. 2:**
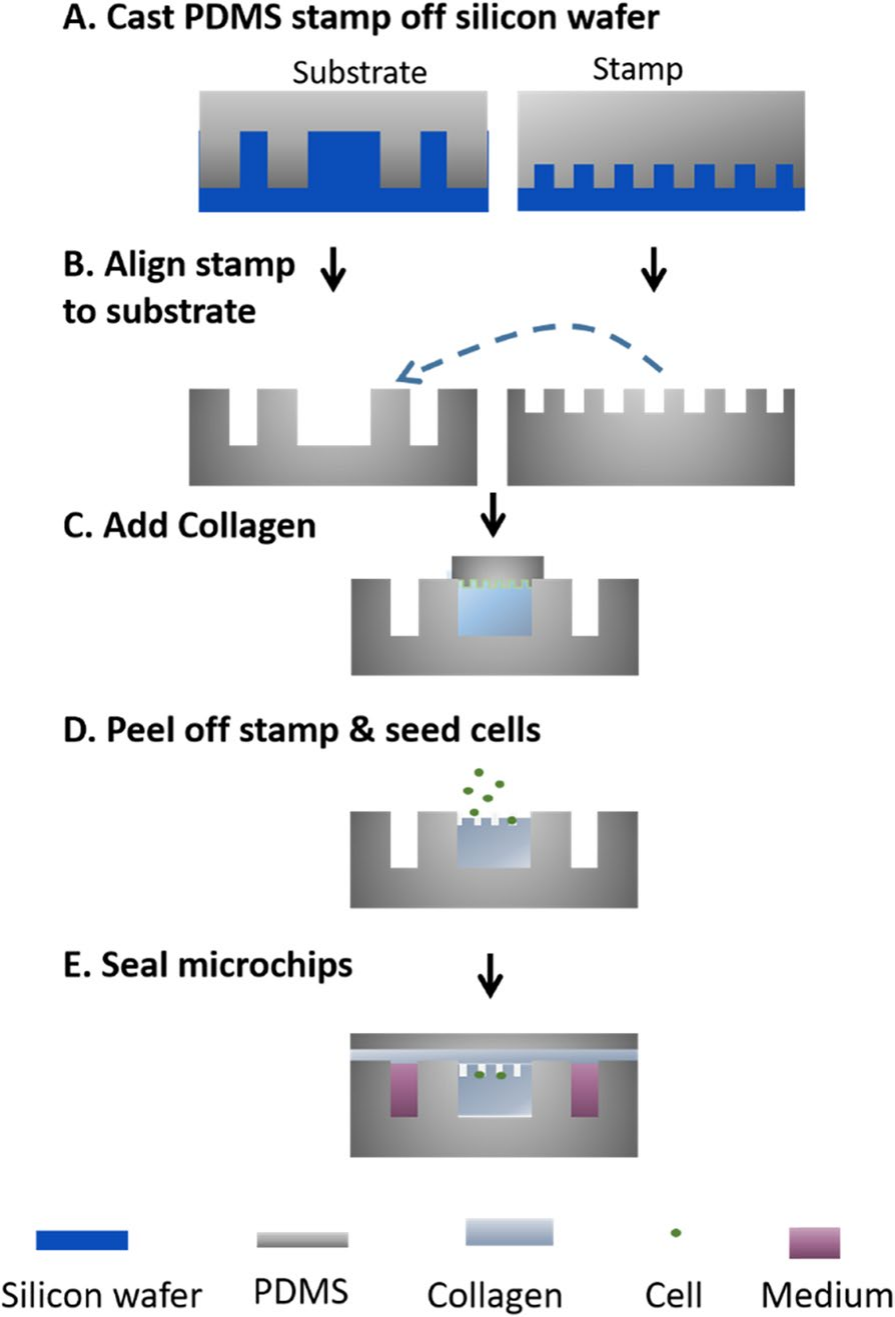
The micro-fabrication process for constructing the 3-D microfluidic *in vitro* model

Therefore, the soft hydrogel micro-fabrication and the microfluidic system was integrated to develop the system suitable for lung cancer cell invasion studies. The structure and size of micro-chambers within the collagen in this 3-D model are also like that of the alveoli in lung (Fig. 1). It has been tested that cell culture medium can perfuse through the whole collagen platform within one hour, thus it can provide enough nutrition for cells. Cancer cells confined in the micro-chambers can invade through the surrounding collagen and reach to the distant region, like the *in vivo* invasion process, then metastasize to other area through blood vessels and forming secondary tumors at last. The fabrication process is described in the Method section and shown in Fig. 2.

The *in vitro* 3-D model has been successfully constructed, and cells are confined in the micro-chambers very well (Fig. 1 and Fig. S1). The images show the regular shape of the fabricated micro-chamber arrays and cells confined within the chambers. There are almost no cells outside of the micro-chambers once the micro-fabrication and cell seeding are completed successfully.

### Cell morphology in the 3-D micro-chamber

The micro-chambers in this 3-D *in vitro* model are located inside collagen hydrogel. Therefore, the substrates for cell culture are all quite soft, and cells can invade out of the micro-chambers by squeezing through the mesh pores of the hydrogel or by degrading the hydrogels with secreted Matrix metalloproteinases (MMPs). In 3-D micro-environment, cells show different morphology from that in 2-D micro-environment (on traditional polystyrene Petri dish substrate).

**Fig. 3:**
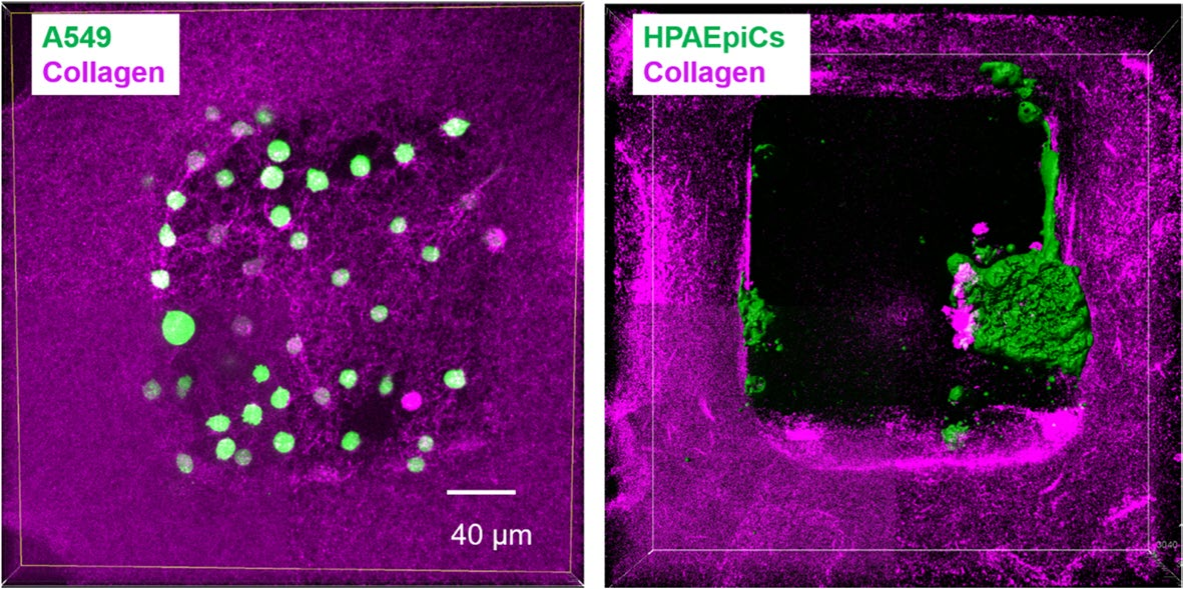
Confocal microscopy images show the cell morphology of A549 and HPAEpiCs in one micro-chamber of the 3-D *in vitro* model. A549 lung cancer cells distributed in the micro-chambers in isolated mode, while normal lung epithelial cells HPAEpiCs can form tissue like assembly within the ECM chamber

In 2-D micro-environment, A549 cells behave normally as other adherent cells, like the normal lung epithelial cells HPAEpiCs, which are both presenting the spindle-like shape (Fig. S2). In contrast, A549 cells in the micro-chamber of the 3-D model present a round shape mostly at the first few days after seeding (Fig. 3 and Fig. S1).

Although the cell assembly mode of A549 and HPAEpiCs are similar on 2-D solid substrate, both isolated spreading on the substrate and forming cell monolayers with higher cell density; their behaviors in the 3-D model perform distinctively variance. As shown in Fig. 3, one day after cell seeding, cancer cells A549 in the micro-chamber of 3-D *in vitro* model presents isolated distribution and round shape phenotype, while the epithelial normal cells HPAEpiCs show highly aggregated tissue-like clusters, and a few isolated cells are also mostly in spindle-like stretched shape. Because cancer cells like A549 lost most of the adhesion ability to other epithelial cells, thus they presented the isolated distribution and can escape from the normal tissue, then realizing the invasion process. In contrast, normal epithelial cells, like HPAEpiCs, keep strong adhesion to the surrounding cells and tissues, thus fulfill their physiological function as a well assembled tissue. This morphology comparison of cancer cells and normal epithelial cells between in 2-D and 3-D micro-environment clearly reveals that this 3-D micro-chamber culture model can better reproduce the *in vivo* micro-environment.

### Cell proliferation within the 3-D micro-chamber model

**Fig. 4:**
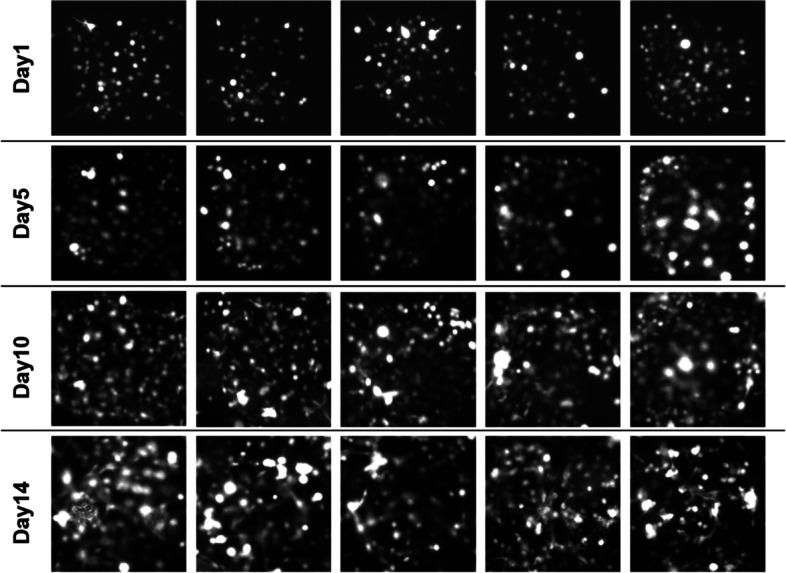
A549 cell growth from Day 1 to Day 14 in representative micro-chambers of the 3-D *in vitro* model

Cell proliferation is one of the fundamental processes for cancer studies. In traditional 2-D model, due to the rapid cancer cell proliferation rate and the limited space, only a short period (around 3–5 days) of cell proliferation can be monitored and analyzed. Then cells must be passaged, and the cell proliferation cycle would be disturbed. In 3-D model, the space for cell proliferation has been expanded dramatically, thus cells can proliferate in 3-D micro-chambers for a much longer time than on 2-D substrate. As shown in Fig. 4, in the micro-chambers of 3-D model, A549 cells kept proliferating on the first 5 days, without much cell morphology change or migration. The cell growth can be observed for as long as 14 days, and cells activity was still quite good.

**Fig. 5:**
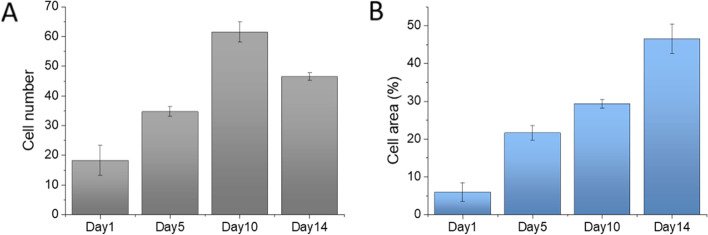
Cell proliferation rate represented with (**A**) cell number and (**B**) cell area percentage

We analyzed the cells in the 3-D models and conducted the statistical research on the cell proliferation rate. Due to the compromise of field of view and the resolution for distinguishing single cells, we defined two parameters, the cell number and cell area percentage, to quantify the cell proliferation individually. Cell number was analyzed by processing fluorescent images and count the cell numbers with software ImageJ. Cell area percentage is the total cell area in each micro-chamber divided by the micro-chamber area, which is also analyzed with ImageJ. Because the cell aggregated at late stage (~ Day 14), it is hard to recognize each cell within the cell clusters. At Day 14, the cell number started to decrease a little bit (Fig. 5A). On the other side, the analyzation of cell area percentage was not disturbed by the cell aggregation issue, so the cell area percentage kept increasing during the whole 14 days, indicating the constant cell proliferation in the 3-D micro-chamber model. Therefore, the two parameters can be considered simultaneously for precising analysis.

### Characterization of lung cancer cells invasion in the micro-chambers

Characterizations of cell invasion are critical for cancer research and the further drug screening tests for cancer therapy. However, the previous methods for characterizing cell invasion are conducted mostly on 2-D substrate, which cannot observe the cell-ECM interaction during the invasion process, thus lacking the reality as *in vivo*. In this 3-D model, cancer cells were confined in the collagen micro-chambers, like the *in vivo* micro-environment of pulmonary gland or alveoli. If the tumor cells are not invasive, cells would grow within the micro-chamber; otherwise, cells would invade through the collagen gel, and escape out of the micro-chamber into the surrounding ECM, either by squeezing through or by degrading the ECM.

**Fig. 6:**
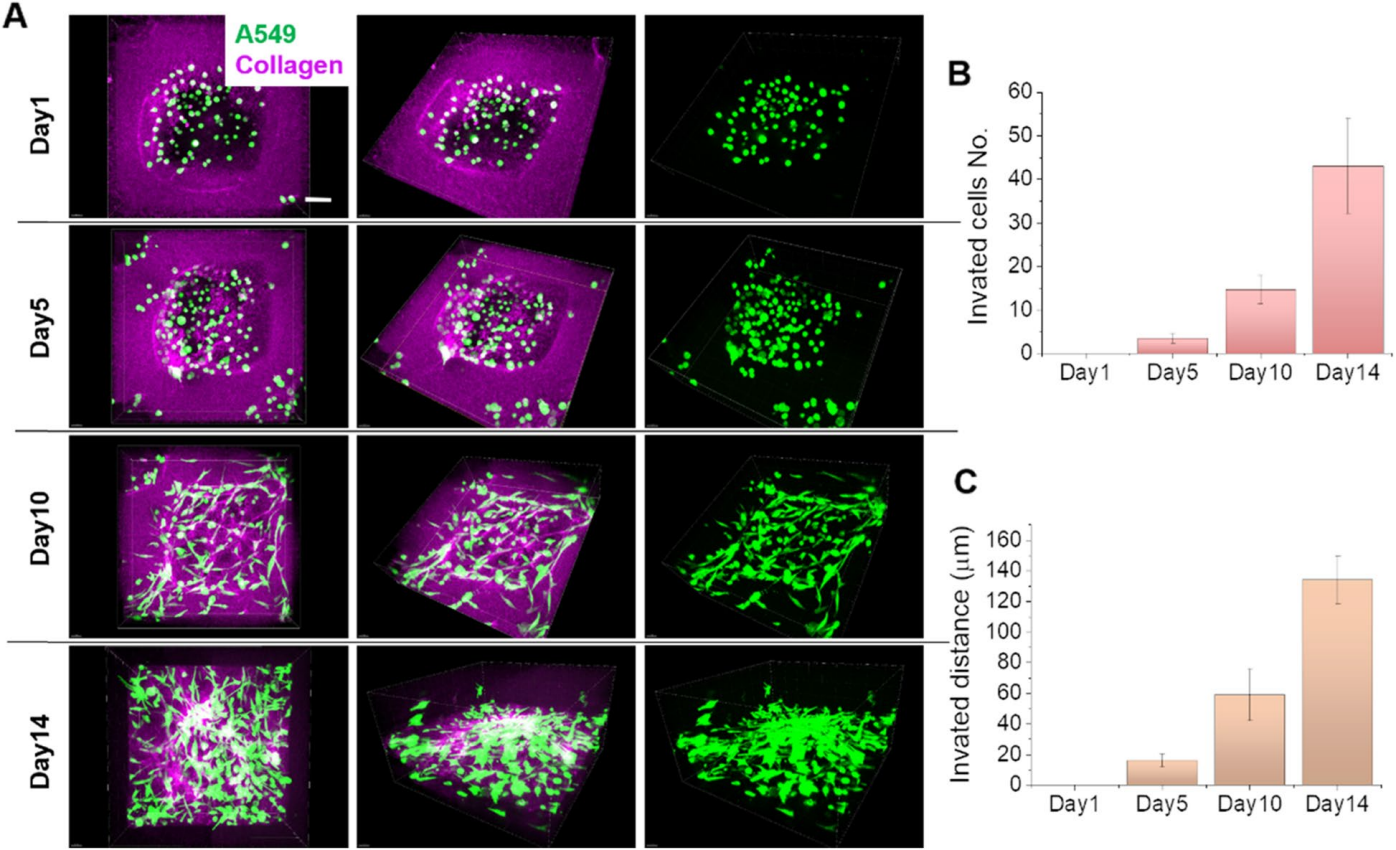
A549 cell invasion in the 3-D micro-chamber of the *in vitro* model. **A** 3-D images of cells cultured in the micro-chamber within collagen on Day 1, Day 5, Day 10, and Day 14. The first two columns are in front view and tilted view, respectively. The third column shows cells channel only. Scale bar is 50 μm. **B** The number of cells invaded out of the micro-chambers on Day 1 to Day 14. **C** The farthest distance that cells invade out of the micro-chambers on Day 1 to Day 14

Non-small-cell lung carcinoma (NSCLC) cells A549 were seeded into the micro-chamber of the 3-D model and the invasion process was analyzed in detail. The representative 3-D images clearly show that on the first 5 days, cancer cells mostly proliferate at the original positions in the micro-chamber (Fig. 4 and 6A). Only a few cells changed the shape to extend short protrusions, while most cells still present round shape and are sparsely distributed. Once the cell density reached to a certain point (on Day 12), most of cells started to transform to the mesenchymal morphology and extend lots of protrusions invading into the ECM. Therefore, the A549 lung cancer cell invasion can be considered as a sudden change, which is an accumulation of cancer cell population, other than continuous cell invasion since cell seeding. In contrast, for some highly malignant and strong invasive cells, such as breast cancer cell MDA-MB-231, once seeding in the micro-chamber of this 3-D model, cells started to extend the protrusions into the surrounding ECM, also called the invadopodia, and finally complete the whole-cell invasion process (Fig. S3). The original micro-chamber structure was also destroyed, indicating the damage of the tissue near the primary tumor site. The reason that cells can extend protrusion into surrounding ECM is probably due to the cell secretion of MMPs, which has been shown to play a major role in regulating invadopodia function [28].

Although the cell morphology during the invasion process has a sudden change, the invasion rate during the whole period kept a constant speed, which can be shown by either invaded cell numbers (Fig. 6B) or the invaded cell distance (Fig. 6C). The 3-D model provided the cancer cells with a more compatible micro-environment, allowing the cancer cells proliferating and invading for a long time, thus more like the process as in vivo.

## Conclusions

An engineered 3-D *in vitro* model for cell culture and invasion studies has been developed with advanced micro-fabrication technique on soft hydrogel within microfluidic system. The 3-D model allows the researcher look into the cells’ interaction with external ECM micro-environment. In this report, the constructed microfluidic platform can provide nutrition and bio-chemical factors in a continuous mode and has the potential to form stable chemical gradients for tumor research. Furthermore, hundreds of micro-chamber arrays can be constructed within the collagen gel, ensuring that all the surrounding micro-environment for tumor cells are natural collagen hydrogel, without the disturbance of the stiff 2-D substrate. In this case, the 3-D *in vitro* model kept a certain void space for the tumor cell culture, like the structure of lung gland or alveoli *in vivo*, other than simply mixing the cells in the hydrogel, which limited the cell motility and cell–cell interaction. Meanwhile, the multiple microchamber arrays can realize high-throughput tests for the cancer invasion studies and even for the future drug screening tests.

In the constructed 3-D micro-chamber model, the cells morphology behaves differently from that on 2-D substrate. It can be seen clearly that cells morphology and assembly mode in 3-D micro-chamber are more like the *in vivo* tissue structures. For example, the lung cancer cell A549 and normal lung epithelial cells have the similar morphology as adherent cells on 2-D substrate, but in 3-D micro-chambers, the A549 cancer cells present isolated distribution, indicating the cancer cells lacking of effective adhesion among cells, while HPAEpiCs present highly assembled structures with strong adhesion just as normal epithelial tissues. Furthermore, compared to the short period of 3–5 days for one passage of cell culture on traditional 2-D culture, the cells can continuously grow and proliferate in 3-D model for more than 14 days without obvious cell damage. In this time period, cell invasion into the surrounding ECM can be observed after the cell density reached to a certain degree. The A549 cell morphology has a sudden change from round shape to the mesenchymal type once they started obvious invasion into the surrounding hydrogel.

It has been observed for the first time that behaviors of cell culture parameters and morphological sudden changes during invasion process in this 3-D *in vitro* model, like that in the *in vivo* micro-environment. In future, the micro-fabricated 3-D *in vitro* model can be considered as an advanced technique for cancer cell studies, especially for the cell invasion research. Because of the highly controllable physical parameters, and the high throughput, the 3-D micro-chamber model can also serve as a promising platform for drug screening tests which target to the cancer invasion pathways.

## Supplementary Information

**Additional file 1**: **Fig. S1**. Overview of the constructed micro-chambers within collagen, and cells pattern in the micro-chambers. (A) Micro-chamber arrays with regular shape, and cells well-confined in them. Dashed squares mark the micro-chamber position. (B) Enlarged view of the micro-chambers and cells in them. **Fig. S2.** Cells morphology of A549 and HPAEpiCs on 2-D *in vitro* culture system. **Fig. S3**. Highly malignant breast cancer cells MDA-MB-231 cells and lung cancer cells A549 cultured in the micro-chambers of the constructed 3-D *in vitro *model. (A) MDA-MB-231 cells started to extend protrusions to invade into the surrounding collagen with high stiffness (high concentration of 6 mg/ml) as early as on Day 1 after seeding. The white arrow indicates one of the protrusions (invadopodia) outside of the micro-chambers. (B) A549 cells in micro-chambers (white dash-line indicating the chamber edge) started to extend protrusions/invadopodia (indicated by the yellow arrows) to invade into the surrounding collagen. The left image taken by laser confocal microscope only shows the cells channel, and the right image shows both channels of cells and collagen fibers.

## Data Availability

The data supporting the findings of this study are available in the supplementary material of this article. The data and materials are available upon request (Qihui Fan, fanqh@iphy.ac.cn).
