# Supplementary material for: Construction of in vitro 3-D model for lung cancer-cell metastasis study
Source: BMC Cancer. 2022 Apr 21;22:438. doi: 10.1186/s12885-022-09546-9 (PMC9027834; doi:10.1186/s12885-022-09546-9)
Supplement: Supplementary file 1 — Additional file 1: Fig. S1. Overview of the constructed micro-chambers within collagen, and cells pattern in the micro-chambers. (A) Micro-chamber arrays with regular shape, and cells well-confined in them. Dashed squares mark the micro-chamber position. (B) Enlarged view of the micro-chambers and cells in them. Fig. S2. Cells morphology of A549 and HPAEpiCs on 2-D in vitro culture system. Fig. S3. Highly malignant breast cancer cells MDA-MB-231 cells and lung cancer cells A549 cultured in the micro-chambers of the constructed 3-D in vitro model. (A) MDA-MB-231 cells started to extend protrusions to invade into the surrounding collagen with high stiffness (high concentration of 6 mg/ml) as early as on Day 1 after seeding. The white arrow indicates one of the protrusions (invadopodia) outside of the micro-chambers. (B) A549 cells in micro-chambers (white dash-line indicating the chamber edge) started to extend protrusions/invadopodia (indicated by the yellow arrows) to invade into the surrounding collagen. The left image taken by laser confocal microscope only shows the cells channel, and the right image shows both channels of cells and collagen fibers. [file 12885_2022_9546_MOESM1_ESM.docx]

**Supplementary Information for**

**Construction of in vitro 3-D model for lung cancer-cell metastasis study**

Rongrong Jiang^1^, Jiechun Huang^1^, Xiaotian Sun^1^, Xianglin Chu^1^, Fangrui Wang^1^, Jie Zhou^2^, Qihui Fan^3*^, Liewen Pang^1*^


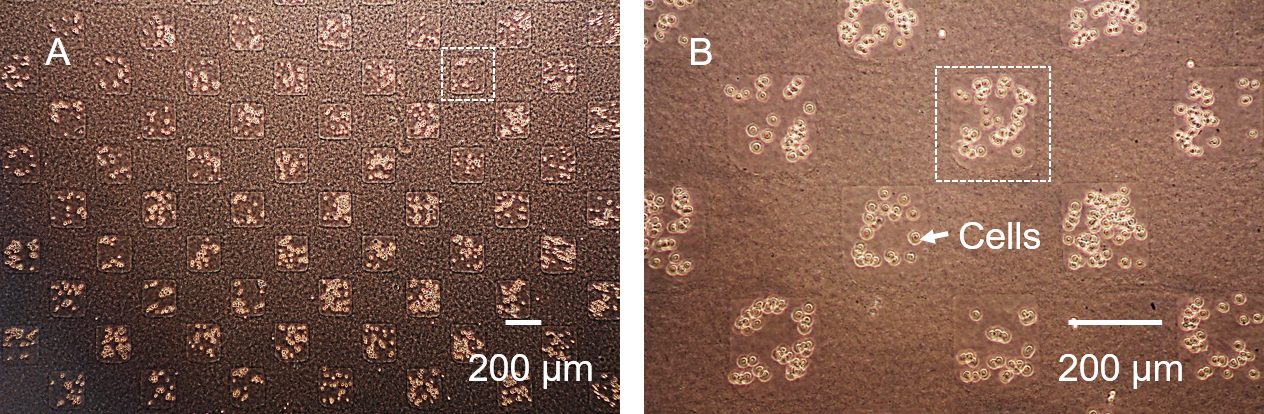


**Fig. S1**. Overview of the constructed micro-chambers within collagen, and cells pattern in the micro-chambers. (A) Micro-chamber arrays with regular shape, and cells well-confined in them. Dashed squares mark the micro-chamber position. (B) Enlarged view of the micro-chambers and cells in them.


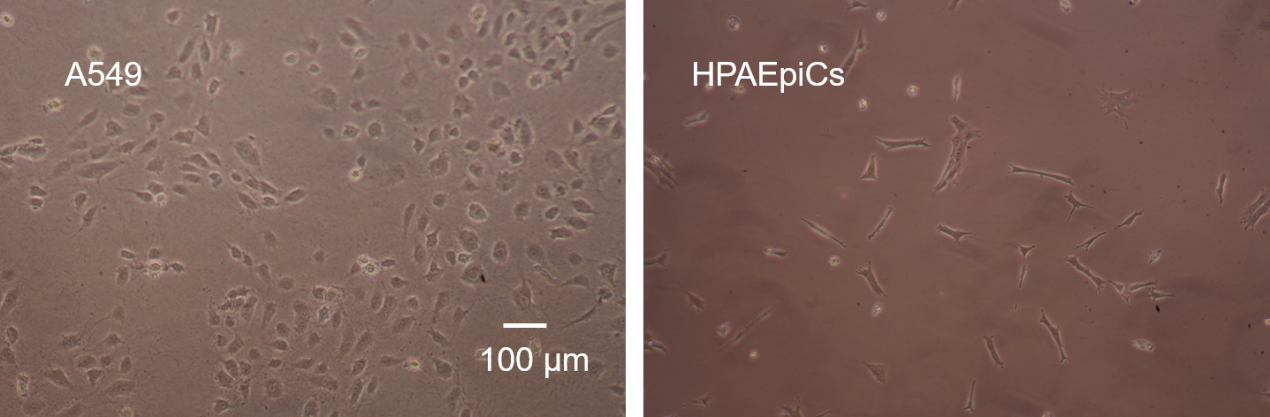


**Fig. S2.** Cells morphology of A549 and HPAEpiCs on 2-D *in vitro* culture system.


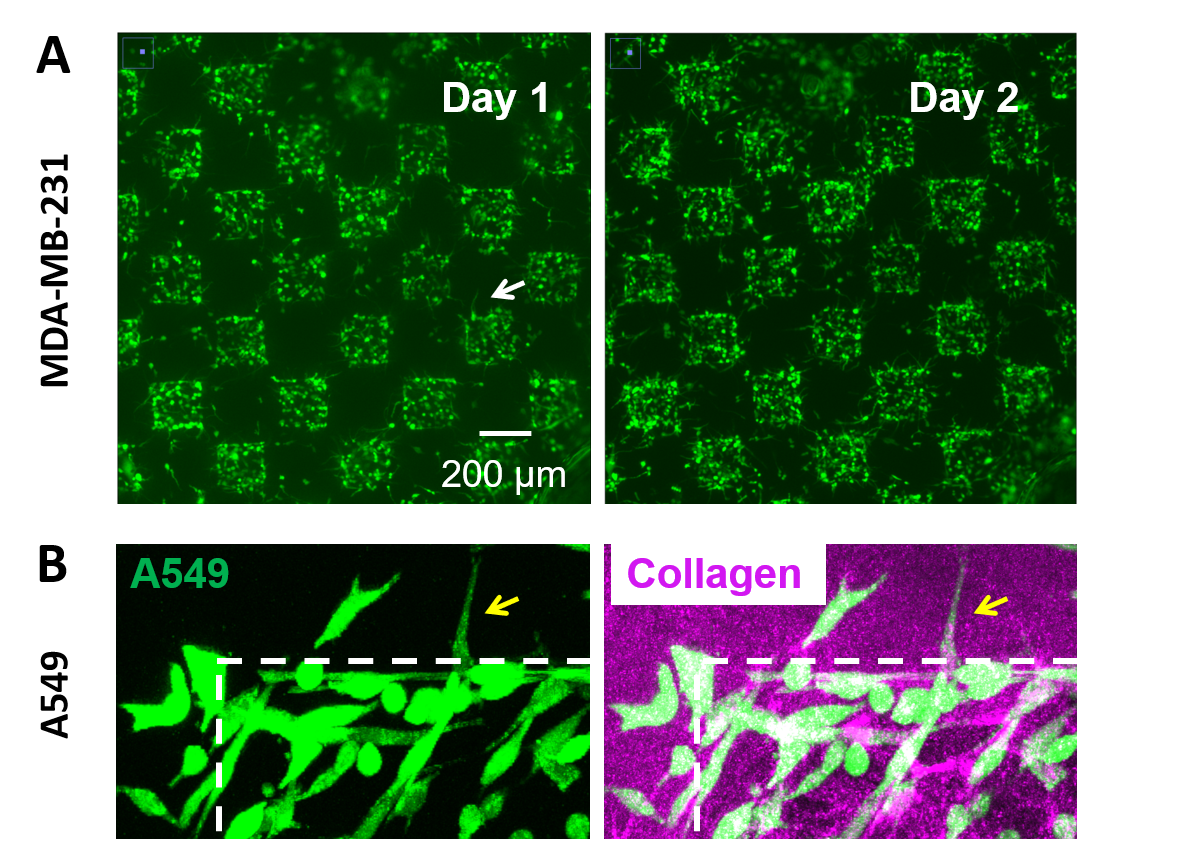


**Fig. S3**. Highly malignant breast cancer cells MDA-MB-231 cells and lung cancer cells A549 cultured in the micro-chambers of the constructed 3-D *in vitro* model. (A) MDA-MB-231 cells started to extend protrusions to invade into the surrounding collagen with high stiffness (high concentration of 6 mg/ml) as early as on Day 1 after seeding. The white arrow indicates one of the protrusions (invadopodia) outside of the micro-chambers. (B) A549 cells in micro-chambers (white dash-line indicating the chamber edge) started to extend protrusions/invadopodia (indicated by the yellow arrows) to invade into the surrounding collagen. The left image taken by laser confocal microscope only shows the cells channel, and the right image shows both channels of cells and collagen fibers.
